# Resilience in Family Members of ICU Patients: Scoping Review of the Literature

**DOI:** 10.3390/jcm14196794

**Published:** 2025-09-25

**Authors:** Sandra Lange, Amelia Dąbrowska, Karolina Koszucka, Wioletta Mędrzycka-Dąbrowska

**Affiliations:** 1 Department of Internal and Pediatric Nursing, Faculty of Health Sciences, Medical University of Gdańsk, Dębinki 7, 80-211 Gdańsk, Poland; langa94@gumed.edu.pl; 2 Faculty of Medicine, Lazarski University of Warsaw, Świeradowska 43, 02-662 Warsaw, Poland; 49175@lazarski.edu.pl; 3 Department of Anaesthesiology Nursing & Intensive Care, Faculty of Health Sciences, Medical University of Gdansk, Dębinki 7, 80-211 Gdańsk, Poland; koszuckakarolina@gmail.com

**Keywords:** family members, resilience, family-centered care, intensive care unit, scoping review

## Abstract

**Introduction:** Hospitalization of a patient in the intensive care unit (ICU) is one of the most stressful events, affecting not only the patient but also their close family members. This situation may lead to the development of anxiety and depressive symptoms, sleep disturbances, and, in some cases, even post-traumatic stress disorder (PTSD). In this context, increasing attention is being paid to the importance of psychological resilience—understood as an individual’s ability to effectively adapt to crisis situations and return to a state of relative emotional balance despite adversity. **Methods:** A scoping review method was used to map terms relevant to the resilience of family members of ICU patients. The aim of this study was to analyze current research on resilience among family members of patients in the intensive care unit (ICU), including measurement tools, facilitating factors, and psychological outcomes. Strict inclusion and exclusion criteria and the PCC framework were used to identify relevant studies. **Results:** The CD-RISC is the most used tool for assessing resilience in the families of ICU patients. Research suggests that resilience is associated with fewer symptoms of depression, anxiety, and acute stress. The studies indicate that spirituality, social/medical staff support, and involvement in care may be crucial factors for maintaining and increasing resilience. Some socio-demographic factors, such as gender, age, and previous mental disorders, may also influence the level of resilience. **Conclusions:** This scoping review highlights the need to implement measures aimed at strengthening the resilience of families of intensive care unit patients by promoting behaviors such as social/staff support and spirituality. Research suggests that levels of resilience may be associated with fewer psychological symptoms in family members of intensive care unit patients.

## 1. Introduction

Hospitalization of a patient in the intensive care unit (ICU) is one of the most stressful events, affecting not only the patient but also their close family members. Relatives often experience intense anxiety, uncertainty, and helplessness caused by the sudden deterioration of their loved one’s health, complex and often incomprehensible medical terminology, and limited access to both information and the patient [1]. This situation may lead to the development of anxiety and depressive symptoms, sleep disturbances, and, in some cases, even post-traumatic stress disorder (PTSD) [2].

In this context, increasing attention is being paid to the importance of psychological resilience—understood as an individual’s ability to effectively adapt to crisis situations and return to a state of relative emotional balance despite adversity [3]. Psychological resilience is a dynamic process shaped by internal personal resources as well as social and environmental support [4]. A high level of resilience among family members of ICU patients is associated with better coping in stressful situations, more effective communication with healthcare professionals, and a reduced risk of mental health disorders [5]. Nurses are ideal to support family resilience. As people who are with patients around the clock, they observe many situations that families of patients in intensive care units have to confront. In a qualitative study by Ellis et al., nurses noted that families faced, among other things, unrealistic expectations regarding the recovery of their loved ones, were overwhelmed by their involvement in patient care, and received conflicting information that caused confusion and anxiety. Using the right approach, nurses can identify and strengthen protective factors for FM resilience [6]. Yu et al., who studied the mechanism of interaction between family functioning, psychological resilience, and illness uncertainty among family members of trauma ICU patients, demonstrated that psychological resilience and family functioning may be protective factors that reduce illness uncertainty [7]. However, family resilience in the ICU has not been thoroughly examined in the literature. To understand this issue more deeply, Sun et al. conducted a conceptual analysis. In their analysis of the concept of ‘family resilience in the ICU’, researchers identified four antecedents and four consequences. The antecedents were as follows: unexpected admission, acute stress, disrupted family order and positive response. The consequences were as follows: the development of resilience, family adaptation, the psychology of the family, and patient support. In addition, the researchers proposed that the implementation of this concept should be based on five key assumptions: identifying family characteristics, helping families establish correct expectations, providing support to help families regain control, promoting family participation in medical decision-making and patient care, and encouraging families to access external resources [8].

### Aim and Rationale

This scoping review aims to analyze current research on resilience among family members of patients in the intensive care unit (ICU) including measurement tools, facilitating factors, and psychological outcomes. The review may contribute to a deeper understanding of the experiences of families facing a medical crisis and serve as a foundation for developing targeted support interventions within the ICU environment. Enhancing the resilience of family members may significantly improve their well-being, the quality of communication with the medical team, and the overall course of patient care during and after intensive treatment.

## 2. Methods

### 2.1. Study Design

A scoping review method was chosen to map concepts relevant to the resilience of family members of ICU patients, as well as to identify gaps in the existing literature and provide directions for future research. The scoping review was conducted in accordance with the methods described in the Joanna Briggs Institute Methodology Manual for Scoping Reviews [9] and PRISMA for ScR [10]. The Arksey & O’Malley framework was used to conduct the scoping review (five steps) [11].

### 2.2. Identifying the Research Question

A scoping review is a type of review that has a broader “scope” with correspondingly less restrictive inclusion criteria. Based on the elements of PCC (population, concept, and context), the following question was posed:

1. Which tools are used to measure the resilience of family members of ICU patients?
2. How does the level of resilience affect other psychological outcomes of family members of ICU patients?
3. Which factors influence the level of resilience of family members of ICU patients?

### 2.3. PCC Framework

Population (P)

Studies that were conducted in patient populations in adult intensive care units were included in the review. We defined an intensive care unit as a hospital ward that provides intensive care to critically ill patients with life-threatening injuries and illnesses. In this scoping review, adults were defined as those who were 18 years of age or older. Family was defined as individuals who are related to patients by blood or marriage. Caregivers/relatives were defined as individuals who accompanied patients during their stay in the ICU (e.g., cohabiting partners, close friends, surrogates).

Concept (C)

The subject of interest was FM resilience.

Resilience “is the process of effectively negotiating, adapting to, or managing significant sources of stress or trauma. Assets and resources within the individual, their life and environment facilitate this capacity for adaptation and ‘bouncing back in in the face of adversity’. Across the life course, the experience of resilience will vary” [12].

Context (C)

The context of resilience in relation to impact on other psychological outcomes (e.g., Stress, anxiety, depression) and what factors influence resilience.

### 2.4. Identifying Relevant Studies

Two authors systematically searched the following databases: PubMed, CINAHL, Scopus, Web of Science. Four databases were used to access a wide range of English-language literature published between 2015 and 2025. The search strategy is presented in Table 1. In addition, the researchers checked the reference list of existing literature reviews to identify eligible articles that may have been missed during the search. The final search was conducted in March 2025.

### 2.5. Study Selection

In the first stage, selection was made by reading the titles and abstracts of articles to identify duplicates and exclude ineligible articles. Inclusion and exclusion criteria are presented in Table 2. Furthermore, the studies had to comply with PCC framework. Any discrepancies were resolved through discussion with the researchers, and at the end of the selection process, full agreement was reached on the articles to be included.

### 2.6. Charting the Data

Two researchers (SL, AD) extracted data. Articles were mapped, extracting relevant information such as author and year, country, study design, aim, population, setting, tool, main findings, and limitations/gaps. The data extraction template has not been piloted prior to its implementation. We charted data from the included articles that matched our review question. Any discrepancies were resolved through discussions between the researchers (SL, AD). In the case of significant disagreements, decisions were made with the supervisor (WMD).

### 2.7. Collating, Summarizing, and Reporting the Results

After charting the data from all included articles to show the most relevant aspects of the review [13], the results were summarized in Table 3. A narrative synthesis was then applied to analyze the results of the included studies [14].

**Figure 1:**
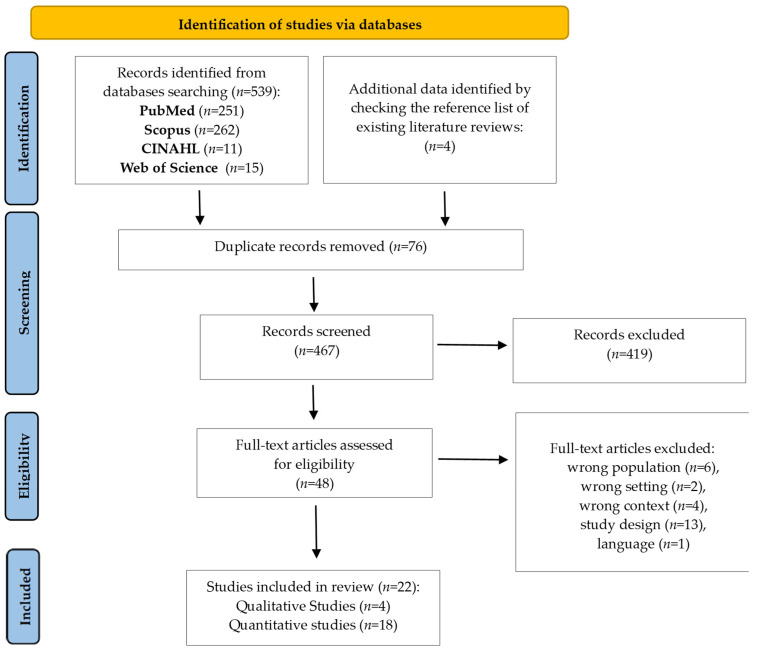
PRISMA flow diagram [15].

The results of the database searches are presented in Figure 1.

**Table 3:** Characteristics of the included studies.

Quantitative Studies								
**Author (Year)**	**Country**	**Study Design**	**Aim**	**Population**	**Setting**	**Tool**	**Main Findings**	**Limitations/Gaps**
Shaffer et al. (2016) [16]	USA	A cross-sectional study	To examine the associations of patients’ and their informal caregivers’ psychosocial resiliency factors with their own and their partners’ emotion domains.	99 informal caregivers 83 dyads of patients n = 87	Neuro-ICU	Resiliency Factors: CAMS-R MOCS-A IBM CSES-R ET4	Greater psychosocial resiliency factors (mindfulness, coping, self-efficacy) were associated with lower emotional distress among both patients and caregivers. However, one’s own resiliency factors were associated with their own, but not their partner’s, emotion domains.	Psychosocial resiliency factors at Neuro-ICU hospitalization relate to patients’ and caregivers’ concurrent and long-term emotional distress.
Sottile et al. (2016) [17]	USA	Cross-sectional study	To describe the association between resilience and PICS-F symptoms.	170 FM	ICU	CD-RISC * HADS IES-R FS-ICU	Resilience is independently associated with less symptoms of depression (14.1% vs. 44.9%, *p* < 0.001), anxiety (14.2% vs. 43.6%, *p* < 0.001), and acute stress disorder (12.7% vs. 36.3%, *p* = 0.001) and greater satisfaction in family members of critically ill patients (76.7 vs. 70.8, *p* = 0.008).	Not assessing long-term psychological outcomes.
Rahmati et al. (2017) [18]	Iran	A quasi-experimental study	To examine the effects of spiritual and religious interventions on the resilience of FM of ICU patients.	34 FM and blood relatives	ICU	CD-RISC *	The findings showed significant effectiveness of spiritual–religious interventions on increasing the resilience of family members of patients.	Small sample size; limited generalizability.
Komachi et al. (2018) [19]	Japan	A cross-sectional study	To determine the level of resilience in family members of patients admitted to the ICU, to verify the relationship between resilience and acute stress symptoms, and to examine the association between resilience and family and patient characteristics.	77 participants	medical/surgical and emergency ICUs	CD-RISC * IES-R	Higher resilience was associated with lower acute stress symptoms among family members of ICU patients. Additionally, certain characteristics, such as being the patient’s spouse and having no prior ICU experience, were linked to higher levels of acute stress symptoms.	Small sample size; limited to Japanese population.
Zale et al. (2018) [20]	USA	A cross-sectional study	To identify associations between resiliency, distress, and caregiver QoL at time of Neuro-ICU admission.	79 informal caregivers	Neuro-ICU	HADS WHOQOL-breve, Resiliency Factors: CAMS-R, MOCS-A; PCS ET4	Mindfulness and preparedness for caregiving emerged as consistent, unique resiliency factors associated with greater caregiver QoL across QoL dimensions. Results highlight the importance of resiliency factors in QoL among Neuro-ICU caregivers and the need for early interventions to support resiliency.	Reliance on self-report measures; sample limited to a single Neuro-ICU setting.
İleri Fikri et al. (2024) [21]	Turkey	Cross-sectional study	To examine sleep disturbances and resilience in the first-degree relatives of patients in a tertiary ICU and to investigate the relationship between sleep quality and resilience.	65 first-degree relatives of critical patients	tertiary ICU	PSQI RSA *	The average score for the RSA was 123.93 ± 24.093 SD—a good level of resilience. No correlation between resilience and sleep quality in the relatives of ICU patients was found. As a result of the evaluation with the PSQI, it was seen that the relatives of the patients experienced high levels of anxiety, depression, and even burnout, and this affected their overall sleep.	Small number of study participants.
Meyers et al. (2020) [22]	USA	Cross-sectional study	To explore the impact of resiliency factors on the longitudinal trajectory of anxiety symptoms in patients admitted to Neuro-ICU and their family caregivers.	103 FM	Neuro-ICU	HADS-A APIM Resiliency Factors: CAMS-R MOCS-A	Mindfulness is interdependent and protective against anxiety in dyads at 3-month but not 6-month follow-up. Higher baseline resilience was associated with a more favorable anxiety trajectory in both patients and caregivers.	Small sample size, limited generalizability, possible self reporting bias.
Vranceanu et al. (2020) [23]	USA	A Randomized Clinical Trial	To determine the feasibility and preliminary effect of the novel dyadic resiliency intervention RT on reducing symptoms of depression, anxiety, and PTS among hospitalized patients and their informal caregivers.	58 dyads of Neuro-ICU patients and their informal caregivers	ICU	HADS PCL Resiliency Factors: CAMS-R MOCS-A DRS	The dyadic resiliency intervention was feasible and potentially efficacious in preventing chronic emotional distress in dyads of survivors of the neuroscience intensive care unit and their informal caregivers. Improvements were sustained through the 12-week follow-up.	Small sample size; preliminary findings require replication in larger trials.
Lin et al. (2022) [24]	China	A cross-sectional study	To identify the level of post-traumatic growth of the family members of neurosurgical intensive care unit patients and to explore its relation to positive personality characteristics, such as gratitude, resilience, and hope	340 FM	Neuro-ICU	PTGI HHI CD-RISC * GQ-6 SSRS	Gratitude, social support, and resilience were significant predictors of PTG.	Measured the PTG of the FM at one time point, without further follow-up.
Papathanasiou et al. (2022) [25]	Greece	A cross-sectional study	To investigate family members’ resilience in correlation with perceived stress and spirituality of coronary, cardiac surgery and general ICU patients.	104 FM	Coronary, Cardiosurgery and General ICU	PSS-14 CD-RISC * DSES	The CD-RISC-25 score ranged from 31 to 100 units with an average value of 70.0 units (SD = 12.9 units). Resilience is significantly correlated with the scales of perceived stress (*p* < 0.001) and daily spirituality (*p* = 0.019). The more FM daily spirituality, the greater their resilience.	Not assessing outcomes after patients’ discharge from the ICU.
Liu et al. (2023) [26]	China	A cross-sectional study	To investigate the impact of COVID-19 infection experience on the mental health status of ICU patients’ family members.	1000 FM	ICU	SDS SAS IES-R PSQI PSS CD-RISC * SCSQ	Family members with a history of COVID-19 infection exhibited significantly higher levels of anxiety, depression, and post-traumatic stress symptoms compared to those without such history. No significant differences in perceived stress, psychological resilience, and coping strategies between the two groups, suggesting that COVID-19 infection may primarily affect participants through neurophysiological mechanisms rather than by altering other factors.	Reliance on self-reported data; potential recall bias.
Wen et al. (2023) [27]	Taiwan	Prospective cohort study	To simultaneously examine and determine co-occurrence of prolonged grief disorder (PGD), post-traumatic stress disorder (PTSD), and depressive symptom trajectories among bereaved family surrogates of ICU decedents.	303 bereaved FM	ICU	PG-13 IES-R HADS	Grief-related psychological symptoms evolved in complex ways during ICU bereavement, as characterized by heterogeneous trajectories. Some ICU bereaved surrogates experienced persistent elevated PGD, PTSD, and depressive symptoms individually or conjointly, underscoring the importance of early screening to identify this population at high risk of comorbid psychological distress trajectories.	Limited generalizability beyond Taiwanese population; reliance on self-report measures; potential recall bias.
Wendlandt et al. (2023) [28]	USA	Prospective cohort study	To identify post-traumatic stress symptom trajectories in family caregivers of patients with acute cardiorespiratory failure and associated factors.	162 family caregivers	ICU	IES-R CD-RISC * SF-36 FIM	Three distinct PTSS trajectories among ICU family caregivers were observed, with 16% of caregivers experiencing chronic PTSS over the subsequent 6 months. Family caregivers with persistent PTSS had lower resilience, prior trauma, higher patient severity of illness, and higher baseline patient functional status compared with family caregivers with persistently low PTSS, with adverse effects on quality of life and work.	Potential selection bias; limited to English speaking participants.
Choi et al. (2024) [29]	USA	An observational, prospective study.	To examine the predictive validity of psychosocial risk screening during admission for caregiver post-traumatic stress disorder (PTSD) at 3- and 6-month post-hospitalization.	99 informal caregivers	Neuro-ICU	PCL-C HADS Resiliency Factors: MOCS-A CAMS-R CSES-R	Baseline PTSD symptoms were the strongest predictor of chronic PTSD at 3 and 6 months. Screening during admission showed moderate sensitivity (75–80%) and high specificity (92–95%) in predicting chronic PTSD. Other factors like anxiety, mindfulness, and bond with patient were associated but did not significantly improve predictive ability.	Limited generalizability; reliance on self-reported measures.
Eugênio et al. (2024) [30]	Brazil	A cross-sectional study	To investigate whether there is an association between hair cortisol concentrations and acute stress symptoms in family members of critically ill patients.	110 FM	ICU	HCC CD-RISC * DUREL IES-R	80% of family members presented with symptoms of acute stress. Only 29 FM (26.4%) were considered resilient. There was no association between intrinsic religiosity and stress symptoms (*p* = 0.721) or between resilience and stress symptoms (*p* = 0.791). A significant correlation was found between high intrinsic religiosity and resilience (rho −0.375; *p* < 0.001).	Potential confounding factors are not fully explored.
Gates et al. (2024) [31]	USA	A prospective observational study	To examine gender differences in PTSS at time of hospitalization, 3 months, and 6 months later among informal caregivers of neurologically intact Neuro-ICU patients, and 2) to explore associations between resiliency factors (i.e., coping, mindfulness, caregiver self-efficacy, intimate care, and caregiver preparedness) and PTSS interacted with gender	92 informal caregivers	Neuro-ICU	PCL-S Resiliency Factors: CAMS-R MOCS-A CSES-R IBM PCS	Resiliency factors such as mindfulness, coping, and self-efficacy were associated with lower post-traumatic stress symptoms. Gender moderated the relationship between mindfulness and post-traumatic stress symptoms, with differences observed between male and female caregivers (e.g., high mindfulness at baseline was associated with lower PTSS in males compared to females at 3 months)	Small sample size; findings may not be generalizable.
Yu et al. (2024) [7]	China	A cross-sectional study	To investigate the current situation and interacting mechanism between family function, psychological resilience, and illness uncertainty in family members of ICU trauma patients.	230 FM	ICU	Family APGAR scale 10-CD-RISC * MUIS-FM	Family function directly negatively affected illness uncertainty and indirectly reduced it through increased psychological resilience. Psychological resilience served as a mediator between family function and illness uncertainty.	Unable to make definitive conclusions About the causality among the three variables in the Structural Equation Model
Fauzan et al. (2025) [32]	Indonesia	A cross-sectional study	To address this gap by investigating the stress levels and coping mechanisms employed by caregivers of ICU patients	50 FM	ICU	PSS FSQ Resiliency Factors: Brief-COPE	84% of caregivers experienced severe stress; 82% had strong coping mechanisms. A strong and significant positive correlation was found between stress level and coping strategies (r = 0.790, *p* < 0.05).	Limited generalizability: cultural context not deeply examined.
Qualitative studies								
**Author (Year)**	**Country**	**Study Design**	**Aim**	**Population**	**Setting**	**Tool**	**Main Findings**	**Limitations/Gaps**
Wong et al. (2017) [33]	Australia	A Constructivist Grounded Theory	To explore barriers to regaining control within family resilience in ICU context.	38 FM	ICU	Semi-structured interview	Families experience loss of control due to uncertainty, inadequate communication, and emotional strain.	Small sample, one setting, lacks generalizability
Wong et al. (2018) [34]	Australia	Constructivist Grounded Theory	To examine role of social networks in enhancing resilience of ICU families.	38 FM	ICU	Interview	Social and peer support is essential to maintaining resilience; staff interactions are crucial.	Same sample reused; not generalizable
Wong et al. (2018) [35]	Australia	Constructivist Grounded Theory	To understand how families create meaning during ICU stay.	38 FM	ICU	Interview	Meaning construction supports emotional strength and resilience under stress.	Same sample reused; not generalizable
Cypress et al. (2023) [36]	USA	An experimental study (qualitative component—thematic analysis)	To explore how the family resilience framework may help understand the relationship between patient- and family-centered care interdisciplinary rounds and the stressors, resources, organizational, and systems context during critical illness.	84 FM	ICU	Thematic analysis	Participants perceived family engagement during interdisciplinary rounds as central to resilience, acting as “synchronizer,” “moderator,” advocate, and therapeutic presence. This engagement aided in providing forewarning and mindfulness of life’s impermanence.	

ICU—Intensive Care Unit; RSA—The resilience scale for adults; FM—Family Members; FS-ICU—Family Satisfaction in the ICU; Neuro-ICU—the Neuroscience Intensive Care Unit; MUIS-FM—The Mishel’s illness uncertainty for family members scale; ET4—Emotion thermometers; RT—Recovering Together; DASS—The Depression, Anxiety and Stress Scale; DAS 7—Dyadic Adjustment Scale—7 item; PHQ-9-Patient Health Questionnaire; MAAS—Mindful Attention Awareness Scale; GAD-7—The Generalized Anxiety Disorder 7; CD-RISC—Connor-Davidson Resilience Scale; Brief-COPE—Brief Coping Orientation to Problems Experienced; PSS/PSS-14—Perceived Stress Scale (14-item); SCSQ—Simplified Coping Style Questionnaire; PSQI—Pittsburgh Sleep Quality Index; HADS/HADS-A—Hospital Anxiety and Depression Scale; IES-R—Impact of Event Scale—Revised; APIM—Actor–Partner Interdependence Model; CAMS-R—Cognitive and Affective Mindfulness Scale—Revised; MOCS-A—Measure of Current Status—Part A; WHOQOL—World Health Organization Quality of Life; PCS—Pain Catastrophizing Scale; HHI—Herth Hope Index; GQ-6—Gratitude Questionnaire-6; PCL/PCL-S(specific version)—PTSD Checklist; PG-13—Prolonged Grief Disor-der-13; SF-36—Short Form Health Survey—36; MINI—Mini International Neuropsychiatric Inter-view; FIM—Functional Independence Measure; SAS/SDS—Self-rating Anxiety/Depression Scale; PTGI—Post-traumatic Growth Inventory; HCC—Hair Cortisol Concentration; DSES—Daily Spiritual Experience Scale; DUREL—Duke University Religion Index; FSQ—Family Support Questionnaire; SSRS—Social Support Rating Scale; CSES-R—Coping Self-Efficacy Scale—Revised; * Tool used for assess resilience.

## 3. Results

### 3.1. Study Selection

A total of 539 records were initially retrieved from the four databases (PubMed, CINAHL, Scopus, Web of Science) and four additional records from the reference lists of existing literature reviews (total = 543). After removing duplicates and screening titles and abstracts, 495 records were excluded. 48 full-text studies were assessed for eligibility. The primary reasons for exclusion were wrong population (n = 6), wrong setting (n = 2), wrong context (n = 4), study design (n = 13), and language (n = 1). Ultimately, the final analysis included a total of 22 articles (Figure 1).

### 3.2. Characteristics of Included Studies

The results of each study are presented in Table 3. All included studies were compliant with the PCC framework. Twenty-two studies employing various methodologies were included in the review: cross-sectional study (n = 12), quasi-experimental study (n = 1), randomized controlled trial (n = 1), prospective study (n = 4), qualitative study (n = 4). The studies were conducted in the United States (n = 9), China (n = 3), Brazil (n = 1), and Turkey (n = 1), Greece (n = 1), Japan (n = 1), Taiwan (n = 1), Indonesia (n = 1), Iran (n = 1), and Australia (n = 3). The studies were conducted in ICUs with varying specializations, including medical, surgical, neuroscience, mixed, and cardiac. The sample size in quantitative studies ranged from 34 people (in a quasi-experimental study) to 1000 people (in a cross-sectional study), and in qualitative studies it was 38 people.

### 3.3. Tools for Measuring Resilience

The most commonly used tool for measuring resilience in research was The Connor-Davidson Resilience Scale. (CD-RISC) [7,17,18,19,24,25,26,28,30]. It consists of 25 items, rated on a 5-point scale (0–4). Higher scores on the CD-RISC scale indicate greater psychological resilience [37]. One research study used its shortened 10-point version—10-CD-RISC [7].

In one study, the Resilience Scale for Adults (RSA) [21], developed by Friborg et al. in 2001, was used [38]. The RSA is a 33-item self-assessment questionnaire used to measure resilience in adults. Respondents rate their agreement with statements on a 7-point Likert scale [39].

Other researchers used tools to measure resilience factors such as Mindfulness—The Cognitive and Affective Mindfulness Scale Revised (CAMS-R) [16,20,23,29,31,40]; Coping—The Measure of Coping Status-A (MOCS-A) [16,20,23,29,31,40]; Brief Coping Orientation to Problems Experienced (Brief-COPE) [32] or Simplified Coping Style Questionnaire (SCSQ) [26]; Intimate care—Intimate Bond Measure (IBM) [16,29,31]; Self-efficacy—Scale for Caregiver Self-Efficacy (CSES-R) [16,29,31]; Emotion domains—Emotion Thermometers (ET) [16,20]; Preparedness for caregiving—The Preparedness for Caregiving Scale (PCS) [20,31]; and dyadic interpersonal interactions measured by the Dyadic Relationship Scale (DRS) [23].

### 3.4. The Impact of Resilience on Other Psychological Outcomes

Soltie et al. demonstrated a significant relationship between resilience levels and symptoms of depression, anxiety, and acute stress. Resilient FM exhibited fewer symptoms of depression (14.1% vs. 44.9%; *p* < 0.001), anxiety (14.2% vs. 43.6%; *p* < 0.001) and acute stress (12.7% vs. 36.3%; *p* = 0.001). In addition, resilience had a positive effect on satisfaction with the care provided (76.7 vs. 70.8; *p* = 0.008) [17]. Shaffer et al. also showed that greater psychosocial resiliency factors (mindfulness, coping, self-efficacy) were associated with lower emotional distress among both patients and caregivers [16]. Wendlandt et al. demonstrated that FM with persistent PTSS had lower resilience, as well as FM with prior trauma, higher patient severity of illness, and higher baseline patient functional status [28]. Similarly, Gates et al. indicated that resilience factors, such as higher mindfulness, higher coping, and higher self-efficacy, were associated with fewer PTSS symptoms at baseline. Differences were observed between male and female caregivers (e.g., high mindfulness at baseline was associated with lower PTSS in men compared to women after 3 months) [31]. A multiple regression analysis conducted by Komachi et al. showed a significant negative relationship between resilience and PTSS (B = –11.98, β = –0.27; *p* = 0.01), which means that low resilience predicted PTSS among FM of ICU patients [19]. Multiple linear regression analysis conducted by Lin et al. showed that gratitude, resilience, and social support were independent predictors of the post-traumatic growth inventory score [24]. Meyers et al. indicated the level of resilience factor (mindfulness and coping) as a prognostic indicator for the pathway of anxiety —with a higher level of mindfulness and coping at baseline was associated with lower levels of anxiety symptoms at all time points (up to 6 months after ICU discharge) [22]. In contrast, Choi et al. did not find mindfulness to be a significant predictor of PTSD pathway. Only the initial PTSD screening was a significant predictor of PTSD at 3 and 6 months [29].

In addition, Yu et al. showed that family functioning has a direct impact on uncertainty about the trajectory of the disease and an indirect impact through resilience. They indicated that resilience and family functioning are protective factors for FM, reducing uncertainty about the trajectory of the disease [7].

Furthermore, a study by Zale et al. showed that resiliency factors such as mindfulness and preparedness for caregiving emerged are associated with greater caregiver QoL, across QoL dimensions. Mindfulness was uniquely and positively associated with Psychological QoL (sr2 = 0.07, *p* = 0.004), and Physical Health QoL (sr2 = 0.12, *p* < 0.001). Preparedness for caregiving was uniquely and positively associated with Social QoL (sr2 = 0.05, *p* = 0.021), Physical Health QoL (sr2 = 0.07, *p* = 0.001), and Environmental QoL (sr2 = 0.14, *p* < 0.001) [20].

However, İleri Fikri et al. did not confirm correlation between resilience and sleep quality in the relatives of ICU patients [21].

### 3.5. Factors Influencing the Level of Resilience

The results of the study by Komachi et al. suggest that age, gender, and history of mental disorders may influence resilience levels—younger FMs are less resilient than older FMs of ICU patients. Females and FMs with a history of mental disorders may have low resilience [19]. Fauzan et al. demonstrated strong and significant positive correlation between stress level and coping strategies (r = 0.790, *p* < 0.05). As stress levels increase, carers are more likely to use coping behaviors, although the effectiveness of these strategies is uncertain [32].

In the study by Eugênio et al., a significant correlation was found between high intrinsic religiosity and resilience (rho −0.375; *p* < 0.001) [30]. The results of Rahamati et al. showed that spiritual and religious interventions are effective in increasing resilience [18]. Another potentially effective intervention was novel dyadic resiliency intervention RT on reducing symptoms of depression, anxiety, and PTS among hospitalized patients and their informal caregivers, analyzed by Vranceanu et al.. The intervention consisted of six sessions: the first two sessions taught basic skills for coping with the trauma of hospitalization and self-care, while the next four were adapted to the individual needs of each pair based on selected modules. The effect persisted during the follow-up visit after 12 weeks. In addition, an intervention-dependent improvement in interpersonal interactions between the two individuals was also observed (0.2 vs. −0.2; difference, 0.4; 95%CI, 0.0 to 0.8; *p* = 0.04) [23].

In qualitative studies, Wong et al. demonstrated that social support and medical staff support are crucial for maintaining resilience [34]. Furthermore, uncertainty, inadequate communication and emotional tension contribute to a loss of control [33]. In turn, constructing meaning (understanding the situation, finding a purpose and contributing to the recovery of loved ones) supports resilience and emotional strength. [35]. Similarly, in the qualitative study by Cypress et al., FMs perceived their involvement in interdisciplinary rounds as crucial for resilience [36].

### 3.6. Research Gaps/Limitations

Overall, there is a gap in the research on the impact of resilience/resiliency factors on the psychological outcomes of FM of ICU patients and the assessment of these outcomes after discharge from the ICU—including lack of follow-up. Furthermore, studies are limited to specific populations, e.g., Neuro-ICU Patients, Japanese population, which limits the possibility of generalizing the results. However, in the case of some studies, it would be appropriate to limit the scope to a specific population (e.g., studies on spirituality/religiousness-resilience). Similarly, there is a gap in research on interventions that could maintain/strengthen resilience levels. In particular, there is a lack of high-quality RCTs. Additionally, it would be worth investigating which socio-demographic characteristics may influence resilience levels.

## 4. Discussion

We conducted a review of the available literature on resilience in families of critically ill patients, focusing on methods of measuring resilience in this population, the impact of resilience levels on FM psychological outcomes, and factors that may influence FM resilience levels. Although resilience is increasingly becoming a subject of interest among researchers, there is still a lack of sufficient research on resilience among FM of critically ill patients. In particular, studies that would explain the cause-and-effect relationship. To our knowledge, this is the first scoping review that synthesizes the current knowledge on the resilience of family members of ICU patients. The family plays a key role in caring for critically ill patients, providing support for their loved ones. The sudden critical condition of a relative is associated with stress, anxiety, and the need to adapt to a new situation. Therefore, understanding the mechanisms of family resilience is extremely important. Research in this area will enable the identification of families’ needs, allow for the creation and implementation of interventions that strengthen families’ ability to cope with demanding situations, and provide them with appropriate forms of psychological support.

This literature review revealed that researchers use different approaches to measure resilience, measuring either resilience or resilience factors such as mindfulness, coping, self-efficacy. The most commonly used scale for measuring resilience among researchers was the Connor-Davidson Resilience Scale (CD-RISC). The scale has been used in various groups: general population sample, primary care outpatients, psychiatric outpatients in private practice, a study on generalized anxiety disorders, and two studies on post-traumatic stress disorder [37]. The second validated scale for measuring resilience was the RSA scale. The tool measures six types of protective factors. It assesses both personal characteristics (self-perception, plans for the future, social competence, orderly lifestyle) and interpersonal characteristics (family cohesion, social resources) that influence resilience [39]. The RSA can be used in various settings [41]. Among the factors of resilience, researchers most often assessed: mindfulness, coping, intimate care, self-efficacy, emotion domains, preparedness for caregiving, and dyadic interpersonal interactions. Similarly to the review conducted by Pauley et al., which focused on resilience in survivors of critical illness, researchers most often assessed resilience factors such as mindfulness, coping [42].

Despite methodological differences between studies, they suggest that there is a link between resilience and the occurrence of anxiety, depression, and PTSD. Lower psychological resilience was associated with a higher incidence of anxiety, depression, and post-traumatic stress disorder. Comparable results were obtained in other study populations. In a published scoping review, among patients who survived an ICU stay, it also suggested an association between low resilience and a higher incidence of anxiety, depression, PTSD, and several other adverse effects that are known components of PICS [42]. Similarly, in a review of cancer patients, it was shown that cancer patients who express greater resilience experience less psychological distress and are more well adjusted to their cancer [43]. Although these results cannot be generalized due to the heterogeneous nature of the study population, stressful events affect not only the patient but also their family members and may indicate that resilience levels may be important for psychological outcomes. Therefore, resilience should be further studied, not only in the patient population but also in their families/caregivers, who play a key role in the recovery of their loved ones. The development of interventions aimed at improving the resilience of ICU patients’ families, coping with crisis, and adapting to the changed situation should become an important part of nursing care. This was also demonstrated in a review by Shao M et al., which aimed to identify the status quo and key influencing factors of family resilience in cancer treatment [44]. However, it is unclear whether the level of resilience can be a prognostic factor for subsequent PICS-F in FM, therefore longitudinal studies are recommended, which allow researchers to conduct multiple observations of the same variables over a longer period.

Admission of a patient to the ICU often causes psychosocial stress for the patient’s family, which is caused, among other things, by the uncertain course of the loved one’s illness. In a study by Gil-Juliá et al., the admission to the ICU generated a great deal and a lot of stress in relatives. Furthermore, it has been shown that uncertainty about the course of the disease (sudden admission, serious condition, risk of death) were rated as the most stressful factors by the families of patients admitted to the ICU [45]. Yu et al. showed that there may be a link between family functioning, psychological resilience, and uncertainty about the disease among family members. Psychological resilience and family functioning may prove to be protective factors for FM, reducing their illness uncertainty [7]. A similar relationship was observed by Qian Li et al., among caregivers of stroke patients. The study showed that the resilience of family caregivers of stroke patients was negatively correlated with illness uncertainty. Illness uncertainty directly predicted family resilience. However, the relationship between illness uncertainty and family resilience was partially mediated by perceived stress [46]. This suggests that the level of illness uncertainty and the stress experienced may be factors that affect the resilience of FM. Therefore, medical staff should implement interventions aimed at strengthening their sense of control over care, reducing illness uncertainty of their loved ones, and reducing perceived stress, which may ultimately improve family resilience.

One study showed a possible correlation between resilience and the quality of life of caregivers. Among the resilience factors examined, mindfulness and preparedness for caregiving were associated with greater quality of life of caregivers [20]. Mindfulness-based interventions have been studied among other patient populations/their families and have shown promise in improving quality of life [47,48,49]. Therefore, it is extremely important to educate patients’ families to build their sense of self-efficacy in caring for their loved ones. In addition, mindfulness training can significantly improve the quality of life of families whose loved ones have been admitted to the ICU. However, no clear conclusions can be drawn because there is too little data on this topic focusing on FM of ICU patients. Therefore, this area requires further research.

The negative effects of having a loved one admitted to the ICU are common and can manifest as anxiety, depression, and post-traumatic stress disorder. Increasingly studies are focusing on identifying the risk factors that may contribute to these negative psychological outcomes [45,50]. The results of the study by Komachi et al. suggest that factors influencing resilience levels may include age, gender, and history of mental disorders. Older family members, females, and FM with a history of mental disorders may have lower resilience. This is in accordance with the study by Luciano Maciel de Souza, which examined factors associated with depression in family members of ICU patients. It was shown that the increase in the prevalence of depression was associated with female sex and previous mental illness. In turn, in this study, age (younger than 40 years) was associated with a high incidence of depression. Risk factors for psychological distress (anxiety, depression, and stress) in the study by Beatriz Gil-Juliá et al. were having steady partner, being a mother, and being female. Younger age and higher education were associated with reduced stress and anxiety [45]. Although many studies suggest that various socio-demographic factors may influence the psychological outcomes of family members of critically ill patients, their role remains unclear. Research in this area is important to identify family members who are most at risk of negative psychological outcomes and who will need interventions to support their resilience.

In addition, this review identified other factors that may support resilience among family members: spirituality, external and internal support. Spiritual-religious interventions in the study by Rahamati et al. proved effective in increasing resilience [18]. Similarly to interventions that focused on teaching patients and their families how to continue through the trauma of the hospitalization and focus on self-care [23]. For some of the FM population, religion can be an important source of strength and hope during events related to their loved one’s stay in the ICU. It is therefore important to consider providing spiritual interventions for family members and respecting the family’s needs, such as prayer and religious rituals. Social support and support from medical staff are crucial for maintaining resilience. Lack of information, unfamiliar ICU environments, way of communicating, and medical equipment predisposed families to lack control and, consequently, to the risk of increased emotional stress [33]. Helping to construct meaning by understanding the situation and finding purpose can support resilience and emotional strength in FM. This was demonstrated by a study by Wong et al. [34]. This suggests that interventions aimed at building the resilience of families of critically ill patients should perceive the family as a whole, whose needs are multi-level and require the involvement of a multidisciplinary team.

Future research should focus on which clinical interventions are feasible and effective in maintaining and increasing resilience/resilience factors. In addition, there is a need to assess the impact of resilience levels on psychological outcomes at various points in time (from admission to the period after discharge from the ICU). Studies should include larger populations and different ICU specialties to be generalized. In addition, researchers should look for socio-demographic factors that may potentially influence resilience levels, e.g., degree of relationship, age, gender, origin, religiosity. In future studies, researchers should pay attention to the precise use of terminology when reporting results, especially with regard to concepts such as post-traumatic stress disorder (PTSD) and post-traumatic stress symptoms (PTSS). These terms should be used in accordance with the measurement tool used in the study. Using these terms interchangeably may make it difficult to compare results between different studies.

## 5. Limitations

This review has several limitations that should be considered. Four databases were used to prepare this review. Although the selected databases are widely used sources of scientific research, the limited number of databases may have affected the scope of the literature searched and resulted in the omission of publications available in other sources (e.g., works published in local journals). In addition, limiting the review to studies published in English may have led to the exclusion of relevant research published in other languages. Another limitation of this review may be the lack of methodological quality assessment of the included studies. However, it should be noted that, according to the JBI guidelines for scoping reviews, an assessment of the quality of the studies is not required. This is because the main objectives of scoping reviews are to determine what has been studied on the topic, to map the available literature, and to identify research gaps that require further study. Furthermore, the protocol for this review was not published or registered a priori, which we also consider to be a limitation. The term used to refer to family members/caregivers also requires clarification. Although at the beginning of the text a distinction was made between family (persons related by blood or marriage) and caregivers/relatives (persons close to the patient, such as cohabiting partners, close friends, surrogates), these terms are used interchangeably in the rest of the text (family members, caregivers, loved ones, relatives). This is due to a broad understanding of the concept of family, which includes significant people involved in the care of the patient, regardless of their formal status.

## 6. Conclusions

This scoping review identified a lack of sufficient research on the impact of resilience on psychological outcomes and factors that may influence resilience levels in the families of critically ill patients. The articles analyzed on resilience in caregivers of critically ill patients revealed that the Connor-Davidson Resilience Scale is the most used tool for assessing resilience in the families of ICU patients. Research suggests that resilience has an impact on depression, anxiety, stress disorders, distress, and PTSD—the higher the level of resilience among families, the fewer the symptoms. Therefore, resilience should be strengthened among families. The studies included in this review indicate that spirituality, social/medical staff support, and involvement in care are crucial factors for maintaining and increasing resilience. Some socio-demographic factors, such as gender, age, and previous mental disorders, may also influence the level of resilience. This review highlights the need to implement interventions aimed at strengthening resilience in the families of ICU patients by promoting behaviors such as social/staff support and spirituality, which can influence resilience. In turn, higher levels of resilience may positively influence other psychological symptoms experienced by FM of intensive care unit patients.

## Figures and Tables

**Table 1:** Search strategy.

PubMed	(“Critical Care” OR “Intensive Care Units”) AND (“Family” OR “Family Members” OR “Relatives” OR “Caregivers”) AND (resilience OR “psychological resilience” OR “psychosocial outcomes” OR “family function”) Results: 251 Limit: years, language
CINAHL	TI (intensive care unit OR ICU OR critical care) AND TI (family OR relatives OR family members OR caregiver) AND TI (resilience OR “psychological resilience” OR “psychosocial outcomes” OR “family function”) Results: 11 Limit: years, language
Scopus	(ALL (intensive AND care AND unit OR critical AND care) AND TITLE-ABS-KEY (family OR relatives OR family AND members OR caregiver) AND TITLE-ABS-KEY (resilience OR “psychological resilience” OR “psychosocial outcomes” OR “family function”)) AND PUBYEAR > 2014 AND PUBYEAR < 2026 AND (LIMIT-TO (LANGUAGE, “English”)) Results: 262 Limit: years, language
Web of Science	(TI = (intensive care unit OR ICU OR critical care) AND TI = (family OR relatives OR family members OR caregiver)) AND TI = (resilience OR “psychological resilience” OR “psychosocial outcomes” OR “family function”) Results: 15 Limit: years, language

**Table 2:** Inclusion and exclusion criteria.

	Inclusion Criteria	Exclusion Criteria
Participants	FM/Caregivers/relatives of adult ICU patients	FM/Caregivers/relatives of adult NICU patients FM/Caregivers/relatives of adult non-ICU patients
Types of evidence source	Observational, prospective, retrospective, RCT, experimental, qualitative studies	Single-case report, cases report, letters to the editor, editorials, commentary, review
Years considered/Time period	All evidence published in the last 10 years, period 2015–2025	Publications prior to 2015
Language	English	Other languages

## Data Availability

The authors declare that the data of this research are available from the correspondent author on request.
